# Human DNA tumor viruses evade uracil-mediated antiviral immunity

**DOI:** 10.1371/journal.ppat.1011252

**Published:** 2023-03-30

**Authors:** Jessica A. Stewart, Blossom Damania

**Affiliations:** Lineberger Comprehensive Cancer Center and Department of Microbiology and Immunology, The University of North Carolina at Chapel Hill, Chapel Hill, North Carolina, United States of America; University of Arizona, UNITED STATES

## Introduction

It is estimated that approximately 15% of tumors worldwide are caused by viruses [1]. These oncogenic viruses are classified as either RNA (RTVs) or DNA tumor viruses (DTVs) [1]. There are two human RTVs: hepatitis C virus (HCV) and human T-cell lymphotropic virus-1 (HTLV-1), and five human DTVs: human papilloma virus (HPV), hepatitis B virus (HBV), Epstein–Barr virus (EBV), Kaposi sarcoma-associated herpesvirus (KSHV), and Merkel cell polyomavirus (MCPyV) [1]. These tumor viruses (TVs) establish lifelong infection and evade host immunity using several strategies. While not all TV infections cause disease, viral modes of established latency and persistence perturb normal cellular processes, sometimes leading to cancer [1]. Particularly interesting are mechanisms to evade uracil-mediated antiviral immunity, which can be detrimental to the host genome.

Uracil is a noncanonical DNA base that can be misincorporated into DNA during replication or chemically introduced through deamination of cytosines in single-stranded DNA, resulting in mutagenic U:G mismatches [2]. These mismatches can happen through spontaneous hydrolysis or enzymatic cytosine deamination by the activation-induced cytosine deaminase (AID)/apolipoprotein B mRNA editing catalytic polypeptide-like family of proteins (APOBEC) [3]. AID and the APOBEC3 (A3) subfamily of proteins function in adaptive and innate immune responses, respectively. AID is a B cell maturation protein [4,5] expressed in B lymphocytes entering germinal centers within lymph nodes. AID activity is restricted to transcription bubbles of expressed immunoglobulin genes to diversify antibody repertoire. Once mature B cells exit germinal centers, AID expression returns to undetectable levels. Interferon signaling and pro-inflammatory cytokines up-regulate A3 proteins [3]. Humans have seven A3 proteins (A3A, A3B, A3C, A3D/E, A3F, A3G, and A3H) that can target RNA, retroviral nascent cDNA, or single-stranded DNA in replication forks [3]. The AID/A3 proteins successfully restrict both RNA and DNA viruses [3], including some RTVs and DTVs [3,6]. However, A3 restriction of RTVs has largely been determined to be deaminase independent [6,7], i.e., not uracil-mediated antiviral immunity. For this reason, RTVs will not be discussed in more detail.

The AID/A3 uracil-mediated antiviral immunity has often been denoted a “double-edged sword,” as these potent viral restrictors can fail to distinguish between host and viral genomes. Thus, the role of AID/A3 proteins in DTV pathogenesis has drawn considerable interest. Here, we review current knowledge of mechanisms by which DTVs evade uracil-mediated antiviral immunity.

## How do uracils antagonize DNA tumor viruses?

**Fig 1:**
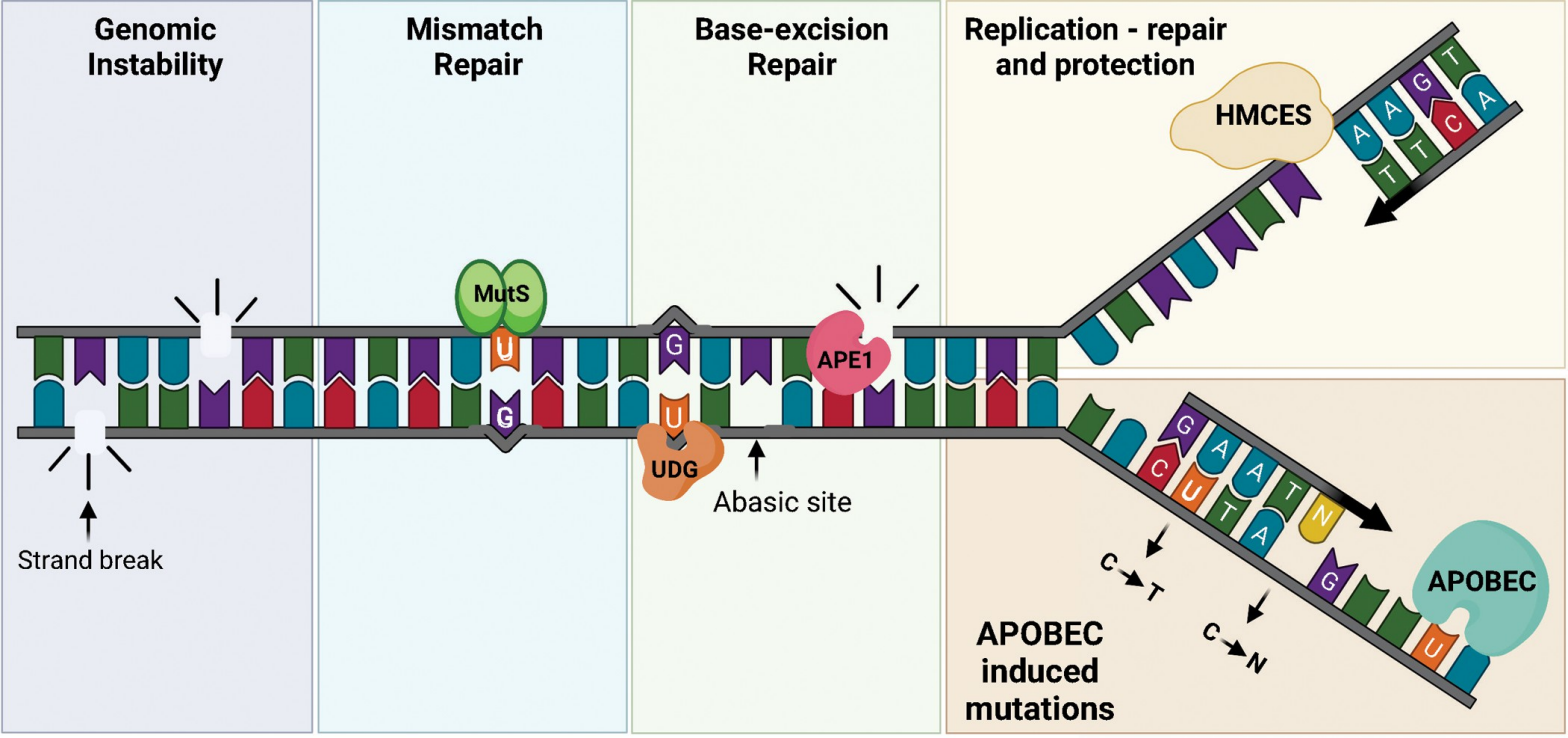
Cellular processing of AID/A3-induced genomic uracils. Schematic of deaminase enzymes targeting single-stranded DNA in the progressing replication fork and the fate of the genomic uracils. AID targets single-stranded DNA in transcription bubbles resulting in U:G mismatches recognized by mismatch repair (MutS; blue panel) or base excision repair (UDG; green panel). UDG excises uracil resulting in an abasic site that is cleaved by APE1. If uracils were present in close proximity and in both strands, cleavage of the DNA backbone by APE1 will induce genomic instability (purple panel). The A3 proteins target cytosines in progressing replication forks. Copying over the resulting uracil will result in a C to T transition mutation (orange panel). UDG can also travel with the replication machinery and excise the uracil, resulting in an abasic site. Abasic sites can be bypassed by translesion synthesis polymerases resulting in a C to N (any nucleotide) mutation (orange panel). Alternatively, HMCES can crosslink the abasic site (yellow panel), halting the progression of replication and protecting the DNA from strand breaks until appropriate repair machinery is recruited. AID, activation-induced cytosine deaminase; APOBEC, apolipoprotein B mRNA editing catalytic polypeptide; HMCES, 5-hydroxymethylcytosine-binding ES cell-specific protein; UDG, uracil-DNA glycosylase; APE1, apurinic/apyrimidinic endonuclease 1.

To understand how genomic uracils promote antiviral immunity, one must first understand the consequences of unrepaired uracils in DNA and how cells manage this noncanonical DNA base in the context of AID/A3 off-targeting to the host genome. In eukaryotic cells, uracils in DNA are typically repaired by the base-excision repair (BER) pathway [2]. BER is initiated through the excision of uracil by a uracil-DNA glycosylase (UDG; Fig 1), leaving behind an abasic site [2]. DNA polymerases may copy over uracil or bypass the abasic site (Fig 1), both of which can result in a DNA mutation. To avoid this, the cell tethers a UDG named uracil-N-glycosylase gene (UNG) isoform 2 (UNG2) to the replication machinery to repair uracils en route [8]. If the resulting abasic site is not immediately repaired, 5-hydroxymethylcytosine (5hmC)-binding ES cell-specific protein (HMCES) crosslinks the abasic site to prevent A3-induced lesions from being processed into double-strand breaks by abasic site endonuclease (APE1; Fig 1) [9]. Finally, if UNG2 or a backup UDG (UNG1, TDG, MBD4, SMUG1) [2] does not remove all genomic uracils, then mismatch repair machinery (MMR) can also recognize and repair U:G mismatches back to C:G pairs [10]. Hence, cells have many strategies to combat uracil mutagenicity and prevent genome instability associated with its repair. Notably, 99% of the human genome (approximately 3,100 Mbp) consists of long noncoding regions of DNA, with only 10% to 15% estimated as functional [11]. In contrast, the majority of viral genomes are coding regions, making uracil genotoxicity a much bigger threat.

**Fig 2:**
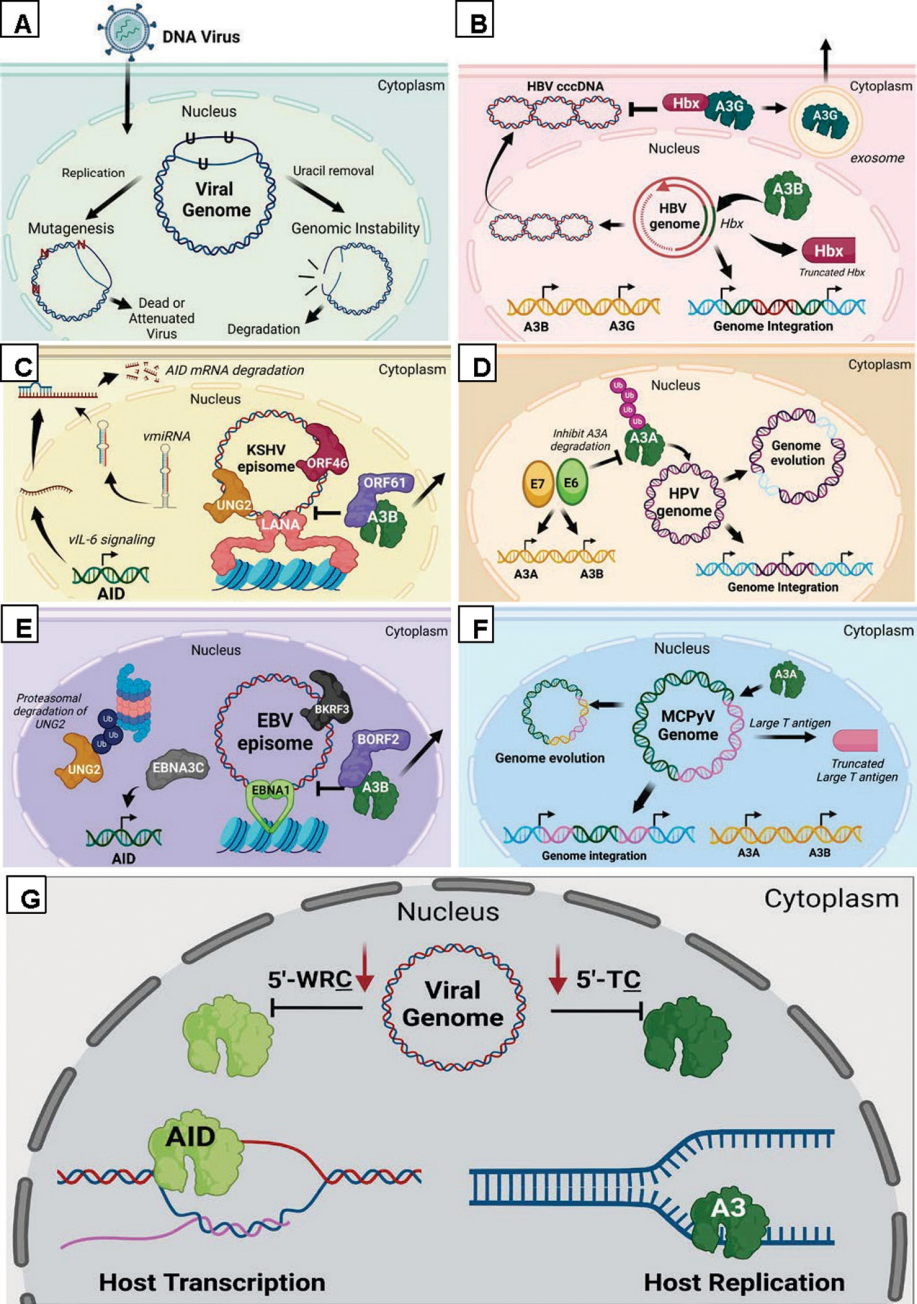
Overview of DNA tumor virus antagonism of uracil-mediated antiviral immunity (UMAI). (A) Representative schematic of successful UMAI by the AID/A3 proteins. Uracils introduced into the viral genome by the AID/A3 proteins are replicated or removed by UDG resulting in a dead or attenuated virus and viral restriction. (B) HBV’s viral protein Hbx antagonizes A3G targeting the viral cccDNA in the cytoplasm, by exosome export. Proposed A3B editing of the Hbx gene results in the carcinogenic truncated mutant and possibly genome integration. (C) KSHV vIL-6 up-regulates AID but antagonizes UMAI through viral miRNA targeting AID transcripts for degradation. KSHV also avoids UMAI by changing nuclear A3's cellular localization via its vRNR protein (ORF61). The KSHV episome may be protected from uracil accumulation during latency because it encodes for its own vUNG (ORF46) or through the tethering of the host UNG2 via LANA. (D) HPV’s oncogenic proteins E6 and E7 keep A3 expression on and inhibit degradation of A3A. It has been proposed that A3 targeting of the HPV genome leads to genome integration and viral evolution, including depletion of 5’ TC dinucleotides. (E) EBV’s EBNA3C protein up-regulates AID but may avoid uracil genotoxicity by degradation of the host UNG2 and utilizing its own vUNG protein (BKRF3). Like KSHV, EBV’s vRNR (BORF2) relocalizes nuclear A3 proteins to alternative cellular compartments. (F) MCPyV, like HPV, may utilize the nuclear A3 proteins for genome evolution and induce genome integration. A3A targeting of the large T antigen has been associated with the carcinogenic truncated protein. (G) Representative schematic of evolved viral genomes (depleted of AID/A3 target sites), which escape UMAI and facilitate tumorigenesis. The persistence of these viruses and chronic infection could lead to constitutive expression of the AID/A3 proteins giving them more opportunity to target the host genome. AID, activation-induced cytosine deaminase; cccDNA, covalently closed circular DNA; EBV, Epstein–Barr virus; KSHV, Kaposi sarcoma-associated herpesvirus; LANA, latency-associated nuclear antigen; MCPyV, Merkel cell polyomavirus; UDG, uracil-DNA glycosylase; UMAI, uracil-mediated antiviral immunity; vIL-6, viral IL-6; vRNR, viral ribonucleotide reductase large subunit; A3A, APOBEC3A; A3G, APOBEC3G.

Compared to humans, DTVs have small genomes (2 to 300 Kbp) and generally do not encode repair proteins to combat enzymatic AID/A3 genomic incorporation of uracil. Gammaherpesviruses are the exception, possessing their own UNG (vUNG) protein [12,13,14]. Uncoordinated uracil removal in DNA leads to persistent abasic sites, which can be processed into strand breaks (Fig 1), which can initiate viral genome degradation [3,15,16], i.e., uracil-mediated antiviral immunity (Fig 2A). Consequently, many human DTVs have developed strategies to evade this uracil-induced genotoxicity.

## What uracil countermeasures are utilized by DNA tumor viruses?

DTVs may survive uracil-mediated antiviral immunity by encoding an AID/A3 antagonist or by hijacking host cellular processes. The latter typically involves the putative UNG2, as it is the most abundant nuclear UDG [17] and the primary uracil repair enzyme [18]. KSHV’s viral latency-associated nuclear antigen (LANA) tethers the cellular UNG2 to its episome (Fig 2), which is necessary for both latent persistence [19] and lytic reactivation [20]. Likewise, HBV’s genome is maintained by UNG2 and is essential to counteract A3-mediated immunity [21]. The LANA functional homolog in EBV, Epstein–Barr nuclear antigen 1 (EBNA1), has not been shown to interact with UNG2. In fact, EBV latently infected cells have an apparent atypical abundance of the mitochondrial UNG isoform (UNG1) over UNG2. This phenotype is exacerbated during lytic reactivation [13] where UNG2 is depleted to undetectable levels while UNG1 levels remain the same [13]. This depletion is synchronous with up-regulation of its own vUNG protein, BKRF3, which is essential for EBV genome replication [13]. This suggests that either the low level of UNG2, or the recently identified nuclear variant of UNG1 [22], may be sufficient to maintain EBV’s genome during latency. Intriguingly, only the DNA-binding domain of the BKRF3 is necessary and the catalytic domain is expendable [13,23]. This observation extends to KSHV’s vUNG, ORF46 [23], suggesting a noncanonical function for these enzymes. This divergence from the host UNG protein function has largely been attributed to an extended DNA intercalating leucine loop, a feature conserved in the murine gammaherpesvirus 68 vUNG, ORF46 [12]. Regardless, the human and murine [14] gammaherpesvirus’ reliance on the vUNGs and host UNG proteins clearly demonstrates the importance of uracil repair to viral persistence.

Antagonism of AID/A3 proteins can prevent the hassle of uracil repair. Therefore, many viruses, including DTVs, perturb transcriptional and/or posttranslational expression of AID/A3 proteins. In gamma- and alpha-herpesviruses, conserved A3 antagonism via the noncanonical function of ribonucleotide reductase large subunit (vRNR) relocalizes some nuclear A3 proteins to other cellular compartments [24] (Fig 2). EBV’s vRNR (BORF2) also inhibits A3B's deaminase activity [25]. The BORF2 domain responsible for this interaction is absent from KSHV’s vRNR (ORF61) [25]. Regardless, the redistribution of the A3 enzymes effectively inhibits deamination of viral replicating genomes in the nucleus [24]. This resembles HBV antagonism of A3G, in which viral protein Hbx lowers intracellular levels of cytoplasmic A3G by cellular export (Fig 2) to avoid damage to the viral covalently closed circular DNA in the cytoplasm [26]. Alternatively, HPV, MCPyV, and KSHV target mRNA expression of AID/A3 proteins. KSHV encodes microRNAs, which down-regulate AID expression during lytic reactivation [20]. In contrast, HPV and MCPyV oncoproteins, E6/E7 and large tumor antigen (LT-ag), respectively, up-regulate expression of A3A and A3B [26,27]. Remarkably, HPV’s E7 protein also inhibits an A3-associated E3 ligase to stabilize intracellular levels of A3A [28] (Fig 2). This unusual strategy has been proposed to promote viral evolution to escape uracil-mediated antiviral immunity [28].

AID/A3-induced uracil-mediated immunity places viruses under selective pressure, so DTVs have evolved to limit A3-preferred cytosine motifs (5′-TC) from their genomes [3,29]. This phenomenon is evident in most DTVs (HPV [30], EBV [29], KSHV [29], and MCPyV [31]). Notably, EBV and KSHV are tropic for B cells and up-regulate AID through viral proteins EBNA3C [32] and interleukin-6 (vIL-6) [33], respectively; consequently, their genomes have underrepresented AID mutational hotspots (5′-WRC (W = A/T, R = A/G)) [3,29]. This DTV evolution has likely resulted from reliance on the NF-κB cell signaling pathway [34], which up-regulates AID/A3 [35] proteins constitutively, putting them under constant threat of uracil mutagenicity. Unfortunately, this can also increase the host genome’s probability of acquiring AID/A3 off-target events (Fig 2) [36,37].

## What are the consequences of viral countermeasures on host genome integrity?

While host cells have many ways to regulate uracil’s genotoxic effect, DTVs perturb these cellular functions using several viral countermeasures. Most detrimental is constitutive AID/A3 protein up-regulation, which can overwhelm cellular uracil repair machinery. Moreover, if one or more uracil host defense systems are compromised by viral countermeasures or genetic predisposition, individuals are at higher risk for developing viral-associated cancers; individuals with UNG gene polymorphisms have been shown to be at higher risk of developing HPV+ cervical [38] and HBV+ hepatocellular carcinomas (HCCs) [39]. It is therefore unsurprising that some viral-associated tumors are enriched for AID/A3 signature mutations, compared to nonviral tumors [40–42].

AID/A3-mediated lesions promote genomic instability in both host and DTV genomes. The resulting strand breaks and repair by nonhomologous end-joining can result in chromosomal aberrations characteristic of many viral-associated tumors. In fact, one common attribute of EBV+ B cell cancers is a chromosomal translocation between immunoglobulin genes (Ig) and proto-oncogene MYC [43,44]. AID is required for Ig-MYC translocations in vivo [45]; thus, it has been hypothesized that EBV’s dysregulation of AID is responsible for this major transforming event [44]. This AID/A3-induced chromosomal instability may also result in recombining virus and host genomes, a phenomenon known as integration, a characteristic of some DTVs (Fig 2) [46]. In several HPV+ tumors, high A3A expression strongly correlates with viral genome integration [28]. Additionally, MCPyV+ Merkel cell carcinomas (MCCs) and HBV+ HCCs harbor most genome breakpoints in the large T antigen [47] and Hbx [48], respectively, which are significantly enriched in A3 signatures [48,49]. These findings indicate that AID/A3 proteins promote viral genome integration, a causal mechanism behind cancer development [46].

While some viral-associated tumors are highly enriched in AID/A3 signature mutations [36,37,40–42], it is sometimes unclear whether these mutations are drivers or passengers in cancer. Passenger mutations do not directly contribute to cancer formation and are often considered coincidental. While A3 signature mutations are found in over half of human cancers [50], many are passenger mutations [51]. However, in viral-associated tumors, evidence exists that A3 mutations drive tumor formation through alterations to viral and host proteins. One primary example is in HPV+ head and neck squamous cell carcinomas (HNSCCs), where the A3 signature is significantly enriched in the PIK3CA helical domain, which is frequently associated with cancer development [40]. Evidence also exists that A3 targeting the MCPyV large T antigen may promote this protein’s truncation, which is a major oncogenic event in MCCs [49]. With better viral-associated cell transformation models and advances in uracil detection technology, such discoveries will likely continue to emerge.

## Summary

Most DTV’s establish persistence in the human host and AID/A3 proteins have not effectively achieved uracil-mediated antiviral immunity and instead are potentially being utilized by DTVs to evolve and promote immune escape. Notably, there are several recent studies suggesting that the A3 proteins are promoting the evolution and viral fitness of SARS-CoV-2 [52,53]. Even more dangerous is the threat to the host genome, which could promote cellular transformation and cancer. For this reason, there is considerable effort toward developing therapeutics to inhibit these proteins [54].
